# Determinants of Consistent Condom Use among College Students in China: Application of the Information-Motivation-Behavior Skills (IMB) Model

**DOI:** 10.1371/journal.pone.0108976

**Published:** 2014-09-29

**Authors:** Zhihao Liu, Pingmin Wei, Minghao Huang, Yuan bao Liu, Lucy Li, Xiao Gong, Juan Chen, Xiaoning Li

**Affiliations:** 1 Institute for Health Education, Jiangsu Provincial Center for Disease Control and Prevention, Nanjing, Jiangsu Province, P.R. of China; 2 Department of Epidemiology and Health Statistics, School of Public Health, Southeast University of China, Nanjing, Jiangsu Province, P.R. of China; 3 Department of Environmental and Global Health, College of Public Health and Health Professions, University of Florida, Gainesville, Florida, United States of America; 4 Department of Medical Statistics and Epidemiology, School of Public Health, Sun Yat-sen University, Guangzhou, Guangdong Province, P.R. of China; 5 Institute of Medical Information & Library, Chinese Academy of Medical Sciences and Peking Union Medical College (CAMS & PUMC), Beijing, P.R. of China; University of Florida, United States of America

## Abstract

**Background:**

Due to the increase incidents of premarital sex and the lack of reproductive health services, college students are at high risk of HIV/AIDS infections in China. This study was designed to examine the predictors of consistency of condom use among college students based on the Information-Motivation-Behavioral Skills (IMB) model and to describe the relationships between the model constructs.

**Methods:**

A cross-sectional study was conducted to assess HIV/AIDS related information, motivation, behavioral skills and preventive behavior among college students in five colleges and universities in Nanjing, China. An anonymous questionnaire survey was conducted for data collection, and the structural equation model (SEM) was used to assess the IMB model.

**Results:**

A total of 3183 participants completed this study. The average age was 19.90 years (SD = 1.43, range 16 to 25). 342 (10.7%) participants of them reported having had premarital sex, among whom 30.7% reported having had a consistent condom use, 13.7% with the experience of abortion (including the participants whose sex partner has the same experience), 32.7% of participants had experience of multiple sex partners. The final IMB model provided acceptable fit to the data (CFI = 0.992, RMSEA = 0.028). Preventive behavior was significantly predicted by behavioral skills (β = 0.754, *P*<0.001). Information (β = 0.138, *P*<0.001) and motivation (β = 0.363, *P*<0.001) were indirectly affected preventive behavior, and was mediated through behavioral skills.

**Conclusions:**

The results of the study demonstrate the utility of the IMB model for consistent condom use among college students in China. The main influencing factor of preventive behavior among college students is behavioral skills. Both information and motivation could affect preventive behavior through behavioral skills. Further research could develop preventive interventions based on the IMB model to promote consistent condom use among college students in China.

## Introduction

China is experiencing a rapid increase in human immunodeficiency virus infection/acquired immune deficiency syndrome (HIV/AIDS). The HIV/AIDS epidemic in China is complex with some populations effected more than others. Currently, sexual contact remains the major route of HIV transmission and this trend continues to increase in China [1], [2]. The changing cultural perceptions of heterosexual sex, combined with limited HIV/AIDS knowledge, have resulted in the increasing prevalence of AIDS and sexually transmitted diseases (STDs) in China [3].

Due to the rapid change in their living environment, lifestyle and relatively privileged access to the outside world through college resources, such as media and the internet, students are becoming sexual active prior to marriage and their initiating sexual maturity earlier than ever [4], [5]. Previous studies have indicated that college students are at high risk of HIV/AIDS infections [6], [7]. Meanwhile, some college students are engaging themselves in high levels of unprotected sexual intercourse with multiple sex partners and other risky sexual behaviors [8], [9]. Thus, research and concerned organizations reach consensus to develop effective HIV preventive interventions among college students [10].

In 2011, 81.6% of new HIV infections cases diagnosed were resulted from sexual transmission (heterosexual and homosexual) in China [1]. To prevent sexually transmitted disease and HIV/AIDS, students are encouraged to reduce risky sexual behaviors and adopted safe sex practices such as condom use. These strategies are supported by previous study as the most cost-effective preventative strategies [11]. In China, schools are the primary locations where students acquire knowledge and behavioral skills. A recent study found that school-based HIV preventive interventions may be the most efficient and methodological strategy for young people at risk of HIV infection [11], [12].

Condom use is one of the most effective methods to prevent HIV/AIDS infection. However, consistent condom use rate is still low in China [5], [13]. The study revealed that only about 40% of college students who have sex experience reported frequent (always/often) condom use in the previous year [5]. To our knowledge, few studies have applied the prevention theoretical frameworks to HIV/AIDS risk behavior prevention, and tested information-motivation-behavioral skills (IMB) model on condom use among college students in China [14]. Under such situation, it is necessary to investigate the predictors of consistent condom use among college students in order to prevent the spread of HIV/AIDS in this population.

The Information-Motivation-Behavioral skills (IMB) model developed by Fisher and his colleagues was designed to predict HIV preventive behavior and necessary elements of HIV preventive intervention. The model has shown that HIV prevention information, motivation and behavioral skills are the fundamental determinants of HIV preventive behavior [15], [16]. The model assumes that an individual must have well-informed information, positive motivation and correct self-efficacy behavioral skills to initiate preventive behavior [15], [17]. Furthermore, HIV prevention information and motivation are generally regarded as independent constructs in the model. Specifically, informed individuals are not necessarily motivated to change their behaviors, and motivated individuals are not necessarily well informed about HIV prevention [18].

The IMB model, compared to other models, has some advantages. 1) It combines the concepts of other theories, such as the self-efficacy concept from the social cognitive theory model and the theory of reasoned action by enhancing the understanding the motivation. The model also considers various types of factors, such as socio-psychological factors that may affect the human behavior [19]; 2) as an integrated multivariable model, it has been proved effective in some people groups, such as high school students [20], unmarried rural-to-urban female migrants [21]; 3) the model can translate theory into practice more easily; and 4) the IMB model are regarded as highly generalizable for HIV risk behavior prevention among various at risk populations [22], [23], [24].

The purpose of this study was to explore the predictors of condom use among college students within the context of the IMB model and to examine the relationships between the constructs of the model. We hypothesized that information and motivation affect preventive behavior largely through behavioral skills, while information and motivation may also directly affect preventive behavior.

## Methods

### Study site

Nanjing, the capital of Jiangsu Province, has approximately 8 million permanent residents and 3 million migrants and is one of the top administrative regions (i.e., provinces, autonomous regions, and municipalities) in China with a highly developed level of economy and education.

### Study population and sampling size

The study was conducted in Nanjing from October to November, 2006. Collaborating with the Provincial Bureau of Education and the Department of Health in Jiangsu Province, the investigators selected institutions that were representative of colleges in terms of student population size and the number of available academic programs. All the invited institutions agreed to participate in the study. Specifically, 5 out of 31 colleges and universities in Nanjing were recruited by using random cluster sampling method.

3238 participants were recruited in the study, which varied from freshman year to senior year. 3183 (98.3%) finished the field survey effectively. Each participant was compensated with a gift for their time and participation at the end of survey.

### Ethics

This study was approved by the ethics committee of Jiangsu Provincial Center for Disease Prevention and Control and school administrators of the selected colleges and universities. Before enrolment in the study, participants were well informed of the objectives, significance, voluntary participation anonymity and confidentiality of this research. Written informed consent was obtained from each participant.

### Data collection

Participants were asked to complete an anonymous questionnaire designed by the program group at the Chinese Ministry of Health for the Health Education of AIDS Prevention. The structured questionnaire included demographic information, such as gender, age, grade, major, hometown, and the constructs of the IMB model, which included prevention information, motivation, behavioral skills, and sexual and reproductive risk behaviors. Participants completed the survey in a private room and the students’ seats were separated from each other for privacy protection.

### Measures

The latent variables included HIV/AIDS prevention information, motivation and behavioral skills which were hypothesized to reflect the constructs of the IMB model. Preventive behavior was used as the main outcome and the dependent variable in the model. Each latent variable was constructed with several associated observable variables that can be directly measured. Each measure of the IMB model constructs was described below.

#### Information

The information was measured with 16 items with “yes”, “no” or “do not know” as answers to assess students’ knowledge on HIV/AIDS. One indicator containing 10 items was related to HIV transmission knowledge (Ia; e.g. “Do you think HIV/AIDS can be transmitted from mother-to-child?”); The other indicator containing 6 items about HIV prevention knowledge (Ib; e.g. “Do you think the correct use of a condom during sexual intercourse can prevent HIV/AIDS infection?”). Positive answers were assigned a score of one while the negative answers or responses of “do not know” received a score of zero. Cronbach’s alpha coefficient for the 10 HIV transmission items and 6 HIV/AIDS prevention items were 0.78 and 0.76, respectively. Individual item scores were summed to produce a total score to represent students’ knowledge on HIV/AIDS. Higher scores indicated participants had access to more information.

#### Motivation

The motivation to practice preventive actions of condom use against HIV/AIDS was assumed to be a function of individual attitudes and relevant subjective norms. It was measured by 5 items constructed from answers on a five-point Likert scale (1 completely disagree and 5 completely agree). The 5 items included the reasons why students had no confidence in persuading their sex partners to use condoms: (Ma; e.g. “fear of being suspected that he or she had sexual experience”); (Mb; e.g. “fear of being suspected of having STDs)”; (Mc; e.g. “suspected that sex partner is suffering from STDs”; (Md; e.g. “fear of being refused by sex partner” and (Me; e.g. “being uncertain of sex partner's feelings”. Cronbach’s alpha coefficient was 0.82 for these 5 items. Higher score indicated a positive attitude towards condom use.

#### Behavioral skills

The behavioral skills were assessed by 4 items from to reflect self-efficacy of asserting condom use. It was measured by 4 items constructed from answers on a five-point Likert scale (1 completely unable and 5 completely able). The 4 items included: (Sa; e.g. “Can you discuss safe sex with sex partner before sex?”; (Sb; e.g. “Will you ask about your sex partner’s sexual experience before sex?”; (Sc; e.g. “Will you apply condoms before sexual intercourse occurs?”; (Sd; e.g. “Can you correctly use a condom during sexual intercourse?”. Cronbach’s alpha coefficient was 0.78 for these 4 items. Higher score indicated more efficient HIV/AIDS preventive behavioral skills.

#### Preventive behavior

Literature review showed that consistent condom use (CCU) usually indicated HIV/AIDS preventive behavior measures which associated with the IMB model [20], [22], [25]. Consistent condom use was calculated by the consistency condom use during premarital sex. It was assessed by the item, “Will you consistently use condom when having premarital sex?”. Responses were given on a five-point Likert scale (1 never and 5 always). Higher score indicated a strong committed consistent condom use.

### Data analysis

Descriptive statistics, including means, standard deviations, frequencies and percentages, were reported. The hypothetical IMB model was examined by the structural equation model (SEM) using the LISREL [26]. SEM compares a proposed hypothetical model that can elucidate a relationship with a set of actual data. The closeness of the variance-covariance matrix implied by the hypothetical model to the empirical variance-covariance matrix was evaluated with goodness-of-fit indices. Fit was assessed with the maximum likelihood chi-square values/degrees of freedom ratio, the comparative fit index (CFI), the non-normed fit index (NNFI), and the root mean square error of approximation (RMSEA), etc [27]. A CFI value greater than 0.95 and a RMSEA value lower than 0.05 indicating a good fit of the model [28]. A non-significant likelihood ratio chi-square test suggests a good fit of the model, however, chi-square is sensitive to sample size, therefore, a χ^2^/df ratio of 3 or less is indicative of an acceptable fit [27], [28].

Confirmatory factor analysis (CFA) was performed with each hypothesized latent construct predicting its proposed manifest indicators. The analysis was conducted to examine the measurement model and the relationships among latent variables and manifest variables [22]. A path model was used to examine predictors of condom use based on the IMB model.

In an effort to generate a parsimonious model, non-significant paths and covariances were gradually dropped until only significant paths and covariances remained. In the trimming of the model, we first reviewed the path coefficients. One non-significant path was eliminated, and the difference χ^2^ was evaluated at each step. Based on the principle of parsimony [29], the initial model had been modified by the modified index (MI = 4). Finally, we obtained a trimmed model that the relationship between motivation and preventive behavior was eliminated. All data underlying the findings were included in the datasets which can be seen in Data S1.

## Results

### Characteristic of participants

A total of 3183 participants (1544 males, 1639 females) completed all measures of the questionnaire (Table 1). The mean age of the participants was 19.90 years (SD = 1.43), and their ages ranged from 16 to 25. Participants from different grade level, the proportions of freshman, sophomore, junior and senior were 25.5%, 24.0%, 28.6% and 21.9%, respectively.

**Table 1:** Socio-economic, demographic characteristics and sexual and reproductive risk behaviors of the participants.

Characteristic variables	N	%
Gender		
Male	1544	48.5
Female	1639	51.5
Age (years)		
16–20	2122	66.7
21–25	1061	33.3
Hometown		
Urban	1988	62.5
Rural	1195	37.5
Grade		
freshman	813	25.5
sophomore	763	24.0
junior	909	28.6
senior	698	21.9
Experience of premarital sex		
Yes	342	10.7
No	2841	89.3
Consistent condom use		
Yes	105	30.7
No	237	69.3
Experience of abortion		
Yes	47	13.7
No	295	86.3
Multiple sex partners		
Yes	112	32.7
No	230	67.3
oral sex		
Yes	218	63.7
No	124	36.3
anal sex		
Yes	31	9.1
No	311	90.9

342 (10.7%) participants reported having had premarital sex, among whom 30.7% reported having had a consistent condom use, 13.7% had the experience of abortion (including the participants whose sex partner has the same experience), 32.7% had experience of multiple sex partners, 4.4% with homosexual behavior, 63.7% had oral sex and 9.1% had anal sex.

### Confirmatory factor analysis

The mean percentages of correct responses to HIV transmission knowledge and prevention knowledge were 86.31% and 71.50%, respectively. It clearly reported that 53.3% strongly supported condom use with sex partner in premarital sex, 53.4% had received HIV/AIDS health education, and that 29.8% had received sex and chastity education, but that 8.7% knew where they could receive voluntary HIV consultation monitoring.

According to K. G. Jöreskog & Dag Sörbom’s method [30], the factor loading of preventive behavior is stable at 1. A preliminary confirmatory factor model estimated the factor structure and relationships among all of the latent variables. Table 2 reports the means, standard deviations, ranges, and factor loadings for the IMB model’s constructs. All the factor loadings in the model were significant (*p*<0.01).

**Table 2:** Summary statistics and factor loadings of the IMB model in confirmatory factor analysis.

Scales		M	SD	FL*
Information				
Prevention knowledge (%correct)	(Ia)	71.50	20.09	0.62
Transmission knowledge (%correct)	(Ib)	86.31	13.23	0.57
Motivation (1–5)				
Fear of being suspected that he/she had sexual experience before	(Ma)	3.66	1.05	0.71
Fear of being suspected of having STDs	(Mb)	4.00	1.01	0.90
Suspect that sex partner is suffering from STDs	(Mc)	3.96	1.02	0.91
Fear of being refused by sex partner	(Md)	3.83	1.00	0.69
Being uncertain on sex partner’s feeling	(Me)	3.56	1.08	0.50
Behavioral skills (1–5)				
Can you discuss safe sex with sex partner before sex?	(Sa)	4.03	1.08	0.66
Will you ask sexual partner’s sexual experience before sex?	(Sb)	3.55	1.12	0.38
Will you apply condoms before sexual intercourse occurs?	(Sc)	3.89	1.04	0.67
Can you correctly use a condom during sexual intercourse?	(Sd)	4.13	0.93	0.71
Preventive behavior (1–5)				
Will you consistently use condom when having premarital sex?	(CCU)	4.32	0.91	-

M = means; SD = standard deviation;

*FL = factor loadings, all significant ≤0.001.

**Figure 1:**
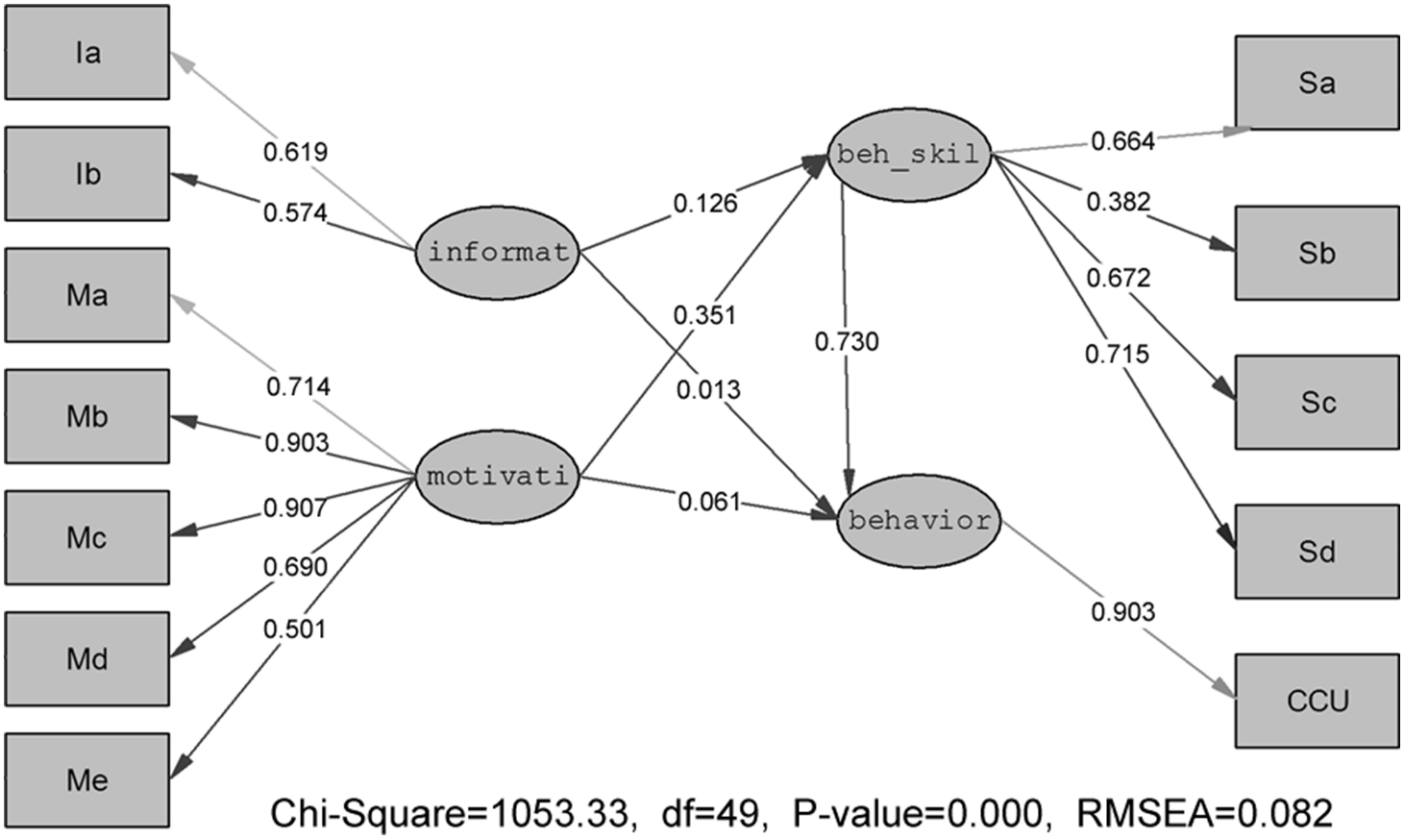
The initial predictive IMB model. Structural equation model depicts regression paths in the IMB model (N = 3183). Large circles represent latent variables; rectangles represent single-item indicators; single-headed arrows represent regression coefficients. Regression coefficients are standardized.

The initial IMB model appears in Figure 1. The fit indices were as follows: χ^2^ = 1053.33, *df* = 49, GFI = 0.946, NNFI = 0.932, CFI = 0.950, NFI = 0.947, and RMSEA = 0.082, which indicated that the initial model should be modified [31], [32].

Table 3 reports the bivariate correlations among the model’s constructs without any directionality of influence among them. The correlations showed that preventive behavior was associated with prevention knowledge, transmission knowledge, motivation and behavioral skills.

**Table 3:** Correlation among model variable.

		I	II	III	IV	V	VI	VII	VIII	IX	X	XI	XII
I	Ia	1											
II	Ib	0.356^++^	1										
III	Ma	0.057^++^	0.069^++^	1									
IV	Mb	0.075^++^	0.095^++^	0.659^++^	1								
V	Mc	0.077^++^	0.086^++^	0.622^++^	0.830^++^	1							
VI	Md	0.066^++^	0.051^+^	0.508^++^	0.589^++^	0.631^++^	1						
VII	Me	0.038*	0.075^++^	0.391^++^	0.413^++^	0.432^++^	0.491^++^	1					
VIII	Sa	0.075^++^	0.063^++^	0.242^++^	0.229^++^	0.229^++^	0.229^++^	0.251^++^	1				
IX	Sb	0.024	0.015	0.124^++^	0.102^++^	0.109^++^	0.109^++^	0.147^++^	0.330^++^	1			
X	Sc	0.082^++^	0.066^++^	0.216^++^	0.188^++^	0.190^++^	0.172^++^	0.259^++^	0.378^++^	0.286^++^	1		
XI	Sd	0.091^++^	0.072^++^	0.195^++^	0.217^++^	0.217^++^	0.212^++^	0.310^++^	0.406^++^	0.224^++^	0.589^++^	1	
XII	CCU	0.093^++^	0.070^++^	0.242^++^	0.259^++^	0.252^++^	0.243^++^	0.290^++^	0.586^++^	0.222^++^	0.390^++^	0.464^++^	1

*p<0.05; ^+^ *p*<0.01; ^++^ *p*<0.001.

### Path model of IMB

**Figure 2:**
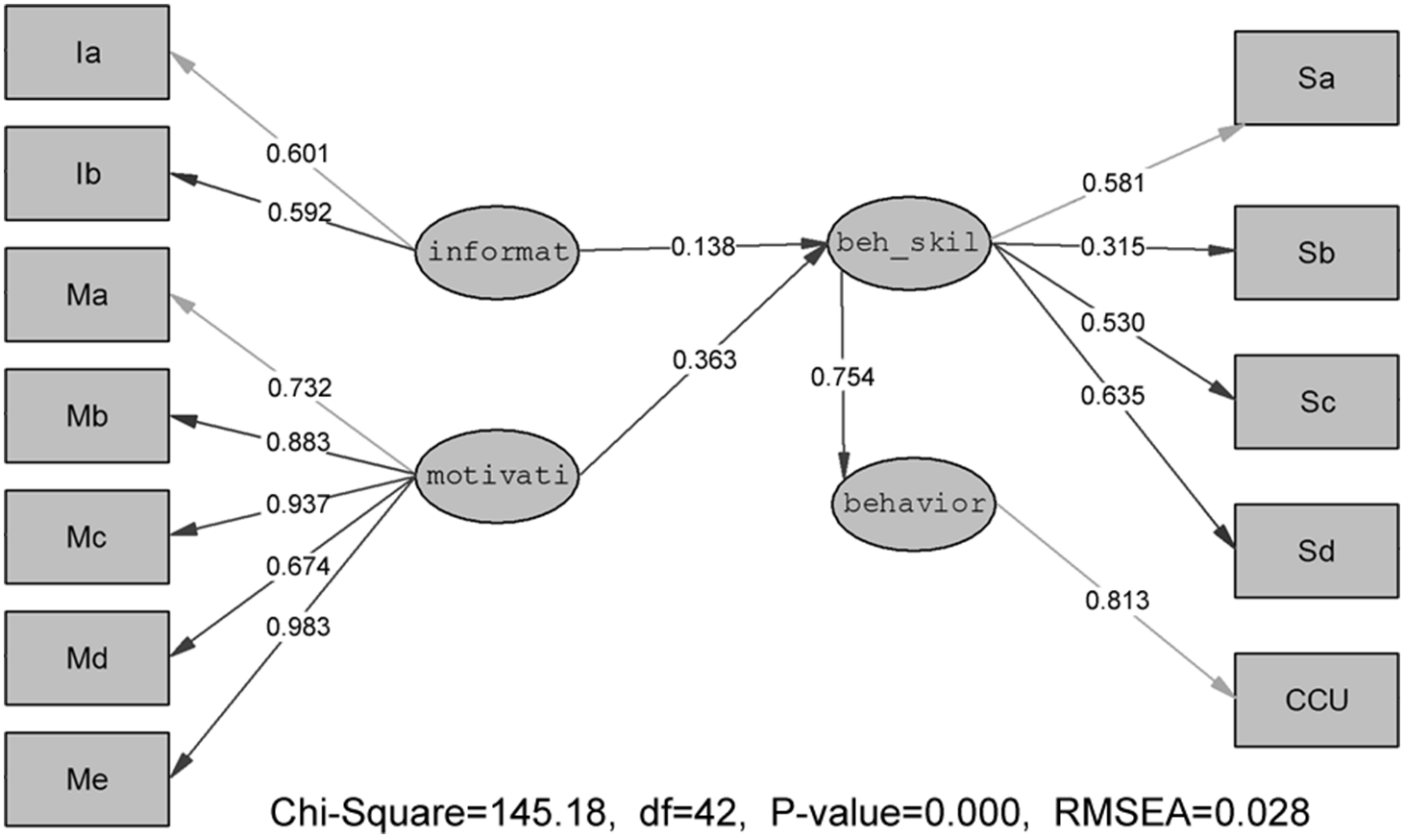
The final predictive IMB model. Structural equation model depicts regression paths in the IMB model (N = 3183). Large circles represent latent variables; rectangles represent single-item indicators; single-headed arrows represent regression coefficients. Regression coefficients are standardized.

The final model is depicted in Figure 2. Non-significant paths and covariances were gradually dropped until only significant ones remained. After modification, the fit indices for the final model were acceptable: χ^2^ = 145.18, *df = *42, GFI = 0.992, NNFI = 0.988, CFI = 0.992, NFI = 0.989, and RMSEA = 0.028. It indicated a-well fitting structure of the final model.

The path coefficient of the final model is showed in Table 4. As expected, a significant predictor of preventive behavior was strongly predicted by behavioral skills (β = 0.754, *P*<0.001), while information and motivation was not significantly associated with preventive behavior. The indirect effects on preventive behavior as reported in the final model were also examined. Information (β = 0.138, *P*<0.001) and motivation (β = 0.363, *P*<0.001) were significantly and positively predicted with behavioral skills, which indirectly affected consistent condom use.

**Table 4:** The path coefficient table of the final model.

Path			regression coefficient	standard regress-ion coefficient	standard Error	T	*P*
Behavioral skills	←	Information	0.150	0.138	0.032	4.644	<0.001
Behavioral skills	←	Motivation	0.365	0.363	0.022	16.577	<0.001
Preventive behavior	←	Behavioral skills	0.878	0.754	0.031	28.528	<0.001
Ia	←	Information	1.000	0.601			
Ib	←	Information	1.082	0.592	0.202	5.369	<0.001
Ma	←	Motivation	1.000	0.732			
Mb	←	Motivation	1.135	0.883	0.025	44.776	<0.001
Mc	←	Motivation	1.212	0.937	0.025	47.654	<0.001
Md	←	Motivation	0.859	0.674	0.024	36.000	<0.001
Me	←	Motivation	1.343	0.983	0.076	17.734	<0.001
Sa	←	Behavioral skills	1.000	0.581			
Sb	←	Behavioral skills	0.447	0.315	0.029	15.437	<0.001
Sc	←	Behavioral skills	0.698	0.530	0.029	24.083	<0.001
Sd	←	Behavioral skills	0.749	0.635	0.027	27.470	<0.001
CCU	←	Preventive behavior	1.000	0.813			

## Discussion

Due to unsafe premarital sexual behaviors, college students often suffer from sexual and reproductive health problems, such as HIV/AIDS, unwanted pregnancy, unsafe abortion, and so on [33]. Related research demonstrated that using condoms in a correct and consistent way can reduce the prevalence of STDs/AIDS, avoid unsafe sexual behavior and prevent unplanned pregnancy, but this type of effect would be reduced if the condom use was inconsistent and incorrect [34].

Recent study showed that high-quality school-based sex education was an effective intervention for HIV-related knowledge spreading and sexual risk behaviors reducing among students, including appropriately delaying the occurrence of sexual debut and significantly increasing the ratio of safe sex [35]. Our results showed that 10.7% college students had premarital sex, which was similar to the findings among two universities in China in 2006 [36], and that some students also had high risk sexual behaviors, therefore, the convenient and targeted behavioral interventions and counseling among college students are necessary.

In the current study, the direct effect of information on preventive behavior was not significant. Our measures were similar to those used in tests of the IMB model with female sex workers [22], juvenile offenders [37], and substance use adolescents [38]. Additionally, some research confirmed the direct effect of information on preventive behavior in tests of the IMB model among women in low-income housing [39] and low levels of HIV-related information [25]. However, our measures demonstrated that the indirect effect of information was significant, medicated through behavioral skills to influence condom use, which was consistent with a study of the IMB model with female sex workers [22]. Some research suggested that information was an important but unnecessary precursor to preventive behavior, especially when the behavior was complicated [16]. The possible reason that information’s effect was sporadic was ceiling effect [40], that is, most of the participants possessed information at a high level, which weakened information’s effect on other factors in the IMB model. In view of the indirect effect of information, it is important and necessary to popularize HIV-related information among college students.

Consistent with the IMB model, in the initial model, condom-related motivation had a direct effect on behavioral skills and preventive behavior [15], [20], [40]. Contrary to the IMB model, in the final model, motivation showed no association with preventive behavior, while motivation also directly predicted behavioral skills and affected preventive behavior through behavioral skills, which is consistent with the study of the IMB model among unmarried rural-to-urban female migrants [21]. Recent study reported that students’ perceptions of preventive behavior affect students’ actual actions in performing preventive behaviors, whether students discussed these issues with their sex partners depended on their perceived susceptibility toward HIV/AIDS [41], [42]. Many students thought that using a condom would affect the sensation of sex then refused to use it [42]. Our measures also demonstrated that “being uncertain of sex partner's feelings” was more likely to influence condom use. The results suggested that the future HIV/AIDS intervention should concentrate on students’ specific perceptions and attitudes on condom use.

As predicted by the IMB model, behavioral skills were significantly associated with preventive behavior, mediated the potential effect of information and motivation on preventive behavior, which was consistent with the previous findings [20], [22], [25]. These findings indicated that behavioral skills played an important role in preventive behavior of this sample. Students worried that serious relationship conflicts could be provoked by discussing safer sex behavior practices with their sex partner, and also they experienced dissonance in viewing their sex partner as a potential health threat. Previous studies reported that the above mentioned students’ worry and dissonance were major obstacles in overcoming the process of behavior change [43]. The results suggested that the importance of engaging in health-promoting behaviors and preventive skills (i.e. higher condom self-efficacy) associated with consistent condom use should be enhanced among college students.

However, it should be cautious to draw conclusions or make generalizations from our findings due to some potential limitations in our study.

A potential limitation of this study is that the cross-sectional research design might limit the ability to establish causal relationships. Because the behavior change is a dynamic process, longitudinal research designs should be implemented to study how behavior changes and provide more effective interventions for the behavior.

The second limitation is the self-reported nature of the questionnaire. Although previous study confirmed that the correlation of self-reported behavior and individual independent evaluation standards was strong and we conducted quality control to reduce potential self-reporting bias, the reliability of the study participants’ responses may be questionable because of the sensitive nature of responses, such as sexual intercourse and condom use [44]. Further studies may verify how accurate the self-reporting method can accurately reflect the IMB level.

Another potential limitation is our measurement of motivation. The concept of motivation is often vague, that is, there is no commonly used operational definition of motivation. Based on Fisher’s conceptualization of the motivational construct [15], [16], [17], we selected five indicators of motivation (“fear of being suspected that he or she had sexual experience before”, “fear of being suspected having STDs”, and so on). However, it is possible that our measurement of motivation may not be relevant for college students.

The final limitation of this study is that there is no direct measure of behavioral skills, such as communication between sex partners on condom use or condom use skills. Instead, we evaluated the students’ willingness to discuss safe sex and sexual experience before sex, condom preparation and correct condom use. Most studies of the IMB model have used self-efficacy as a proxy for behavioral skills. The correct use of a condom plays an important role in preventing HIV prevention; however, the ability to behave assertively with a sex partner may be more relevant for actual condom use [39].

## Conclusions

The results of the study demonstrate the utility of the IMB model for consistent condom use among college students in China. The main influencing factor of preventive behavior among college students is behavioral skills. Both information and motivation can affect preventive behavior through behavioral skills. The relationships among the constructs of the IMB model are also examined. Further research could develop preventive interventions on the bases of the IMB model to promote consistent condom use among college students in China.

## Supporting Information

Data S1 **The raw data for application of the Information-Motivation-Behavior Skills (IMB) Model.**
(SAV)
